# Molecular Pathology of Pancreatic Cancer: From Bench-to-Bedside Translation

**DOI:** 10.2174/138945012800564103

**Published:** 2012-06

**Authors:** Vincenzo Corbo, Giampaolo Tortora, Aldo Scarpa

**Affiliations:** 1 ARC-Net Research Centre, University Hospital of Verona, Verona, Italy; 2 Department of Pathology and Diagnostic, University Hospital of Verona, Verona, Italy; 3 Dipartimento di Medicina, Sezione di Oncologia Medica, Università di Verona, Verona, Italy

**Keywords:** Cancer, diagnosis, markers, pancreas, predictive, prognosis.

## Abstract

Pancreatic ductal adenocarcinoma (referred here as pancreatic cancer) is a lethal disease with the worst prognosis among all solid tumors. Surgical resection represents the only hope for cure but it is possible only in patients that present with local disease (about 20% of cases). Whether dismal prognosis of pancreatic cancer is a result of late diagnosis or early dissemination to distant organ is still a debate. Moreover, this disease shows an intrinsic chemotherapeutic resistance that has been mainly ascribed to the presence of a dense stromal reaction that significantly impairs drugs delivery. Clinical management of pancreatic cancer patients relies on few molecular markers (e.g., the diagnostic marker CA19-9) that, however, present several limitations to their use. The clinical usefulness of somatic alterations in well-characterized genes (such as *KRAS *and *TP53*), whose detection is technically feasible in different biological samples, has been extensively investigated leading to inconsistent results. Furthermore, none of the candidate molecular markers identified in recent years has shown an appropriate clinical performance and therefore none is routinely used. This depicts a scenario where the identification of novel and effective clinical biomarkers is mandatory. Very recent genome-wide comprehensive studies have shed light on the high degree of genetic complexity and heterogeneity of the pancreatic cancers. Although far from being introduced into the clinical settings, results from those studies are expected to change definitively the perspective through which we look at the clinical management of pancreatic cancer patients towards a personalized cancer medicine.

## PATHOLOGICAL TYPING AND STAGING

Ductal adenocarcinoma is the most common malignant tumor of the pancreas, and it is often referred as pancreatic cancer [1]. Pancreatic cancer is an extremely infiltrative neoplasm that usually presents with vascular and perineural invasion in surgically resected tumors. Metastases to lymph nodes, liver and distant sites are all too common [2]. 

The vast majority of pancreatic cancers locate in the head of the pancreas (65%). These tumors tend to present earlier with obstructive jaundice and pancreatitis. Pancreatic cancers are also located in the body (15%), in the tail (10%), or present as multifocal (2%). Tumors of the body and tail tend to present late and are associated with a worse prognosis [3]. Pancreatic ductal adenocarcinoma primarily exhibits a glandular pattern with duct-like structures and must be distinguished from carcinomas of the intrapancreatic bile duct, ampulla of Vater or duodenal mucosa as these tumors have a better prognosis. The term “peri-ampullary cancer” is often used for those tumors arising in the head of the pancreas, and for which there is no possibility to distinguish the tissue of origin [3]. Moreover, several histologic variants of pancreatic cancer have been described, including: adenosquamous carcinoma, colloid carcinoma, undifferentiated carcinoma, medullary carcinoma, signet-ring cell carcinoma, and undifferentiated carcinoma with osteoclast-like giant cells [2]. Some of these variants (such as adenosquamous carcinoma) have a worse prognosis than infiltrating ductal adenocarcinoma and, therefore, need to be identified [2]. 

**Fig. (1):**
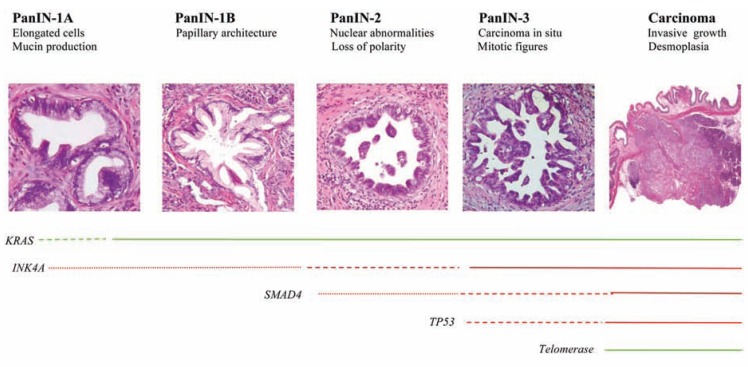
**Precursor lesions of pancreatic cancer: pancreatic intraepithelial neoplasia (PanINs).** PanINs represent progressive stages of neoplastic growth that precede the onset of the invasive carcinoma. The progression from low-grade lesions to carcinoma (from the left to the right) is associated with an increasing number of genetic alterations. Lines represent the stage of onsent of these alterations; the thickness of the line indicates the frequency of the alteration; whereas the colour corresponds to the type of alteration (green, activation; red, loss of function).

Several studies have described three distinct precursor lesions of pancreatic cancer: pancreatic intraepithelial neoplasia (PanINs), mucinous cystic neoplasms (MCNs), and intraductal papillary mucinous neoplasms (IPMNs) [4-6]. Of these, PanINs represent the most frequent and well-characterized premalignant lesions. Histologically, PanINs are classified into PanIN-1, PanIN-2, and PanIN-3 lesions, depending upon the degree of cytologic and architectural atypia [4, 5]. A PanIN to pancreatic cancer progression model has been proposed based on the results of different molecular studies showing an increasing number of genetic alterations in higher grade PanINs (summarized in Fig. **1**) [7]. 

Pancreatic cancer is staged according to the most recent edition of the American Joint Committee on Cancer tumor-node-metastasis classification (Table **1**), which is based on the assessment of resectability by means of contrast-enhanced multidetector computed tomography (CT) [8]. This technique represents the imaging procedure of choice for the initial evaluation of pancreatic cancer [9]. Although tissue diagnosis and pathological staging are essential to determine the most appropriate treatment and prognostic groups, the vast majority of cases (~85%) are represented by non-resectable pancreatic cancers. In these cases the diagnosis is based on histopathological examination of biopsies or cytological specimens and only a clinical staging is possible.

As said above, contrast-enhanced multislice CT scan is the preferred method for non-invasive staging of pancreatic cancer and predicts surgical resectability with up to 90% accuracy [10]. However, a tissue diagnosis is required when unresectable disease appears to be present (e.g., to confirm CT evidence of liver metastasis), and a treatment approach other than resection will be recommended. The preferred methods to obtain tissue for diagnostic purposes is the endoscopic ultrasonography-guided fine needle aspiration (EUS-guided FNA). This technique is highly specific (99%) in the hands of experienced cytopathologists and only rare complications, including severe pancreatitis and seeding of the needle tract with cancer, have been reported after FNA of pancreatic cancer. Other methods include: preoperative endoscopic retrograde cholangiopancreatography (ERCP) brushing for cytology that can be used in cases undergoing endoscoping stenting [11]; and, in selective cases, laparoscopy that can improve staging determining accurately meta-static and vascular involvement [12]. 

**Fig. (2):**
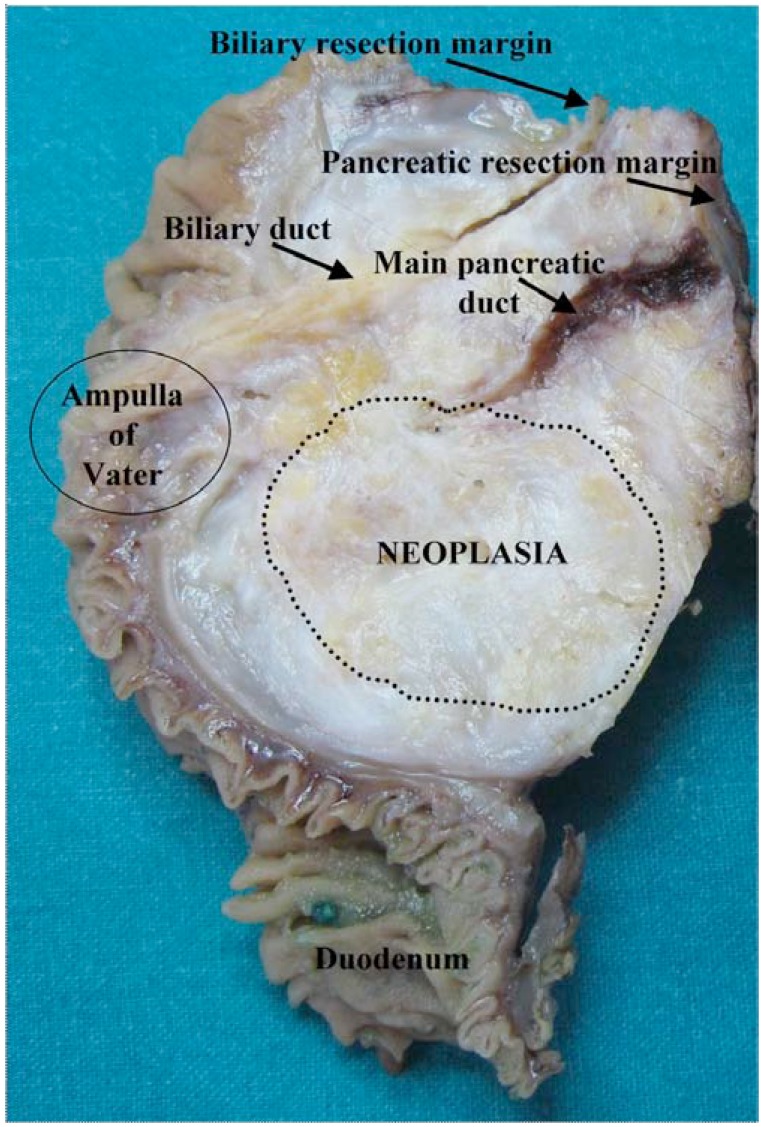
**Gross appearance of pancreatic adenocarcinoma.** The neoplasia (dotted line) presents vanishing borders, infiltrates the major pancreatic duct (square) causing upstream dilation, and gets close to biliary duct (star), without macroscopically involving it. The retroperitoneal margin is posterior.

Poor prognostic factors for pancreatic cancers include high tumor grade, lymph node status, and a positive margin of resection [13-15]. The microscopic resection margin status is an important survival factor [16]. The resection margins (R) are three: pancreatic, biliary, and retroperitoneal (Fig. **2**). Biliary and pancreatic margins are usually evaluated intraoperatively by microscopic examination of cryostatic sections to permit enlargement of resection if they are involved. Retroperitoneal margin is constituted by adipose tissue containing the lymph nodes of the superior mesenteric artery. This is the preferential draining station for most pancreatic neoplasms. Unfortunately, the retroperitoneal margin can be evaluated only after formaline fixation, so it is the real “hot point.” When involved by the neoplasm (R1), the probability that residual neoplasm causes loco-regional recurrence is high and this influences prognosis and survival. Specifically, a positive microscopic resection margin (R1), defined as at least one cancer cell within 1 mm of any surface of the resected specimens, is related to the histological grade and lymph node status rather than tumor size. Furthermore, the results of a very recent work suggest that a margin clearance of more than 1.5 mm is important for long-term survival in a subgroup of patients and that stratification of patients with this criterion may be used to identify those subject that may benefit from adjuvant radiotherapy [17]. 

The poor prognosis of pancreatic cancer is mainly attributed to the inability to diagnose the disease at early resectable stage, to its propensity to disseminate to distant site, and to its resistance to systemic therapies. Improvements in diagnosis, prognosis and treatment of pancreatic cancer have been achieved through the identification of novel molecular markers and targets as well as by the recognition that other cell types other than neoplastic cells are critical components of this complex and heterogeneous disease. In recent years, several serum and tissue-based markers have been proposed for pancreatic cancer. In this review, we mainly focus on the current status of these markers particularly those in the areas of early diagnosis, giving also an insight into the diverse cellular component of pancreatic cancer. 

## MOLECULAR PATHOLOGY OF THE CELLULAR COMPONENTS OF PANCREATIC CANCER 

**Fig. (3):**
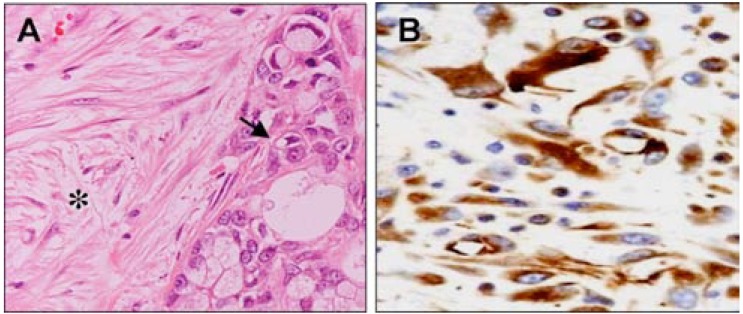
**Cellular components of Pancreatic Cancer.** H&E and immunohistochemical-stained sections of pancreatic cancer tissues: (**A**) Ductal adenocarcinoma composed of epithelial neoplastic cells (arrow) embedded in a fibrous stroma (asterisk); (**B**) Immunostaining of Pancreatic stellate cells with SMA antibody (alpha-smooth muscle actin).

Pancreatic cancer is a complex and heterogeneous entity that is composed of neoplastic cells and its microenviroment (Fig. **3**). The majority of neoplastic cells express a specific pattern of immunohistochemically detectable markers that include: Cytokeratins (Cytokeratin 7, 8, 13, 18, and 19); Carcinoembyonic antigen; Carbohydrate antigen 19-9 (CA19-9); B72.3 (TAG-72); CA-125; and DUPAN 2 [2]. Most pancreatic cancers also express a number of mucins (including MUC1, MUC3, MUC4, MUC5AC), and more recently described markers such as claudin 4 and claudin 18, several S-100 proteins, mesothelin and prostate stem cell antigen [2]. 

Several of these epithelial markers may be useful in differential diagnosis of pancreatic cancers in case of cytologic or histologic metastatic sample. However, no specific immunohistochemical marker exists for pancreatic cance. A potential diagnostic panel should include the markers described above, whose expression define a pancreatic tissue origin as well as markers whose expression is limited to a specific organ other than pancreas, such as the intestinal differentiation marker CDX2 or the pulmonary marker TTF1. An additional feature suggesting a pancreatic origin is the immunonegativity of DPC4 (Deleted in Pancreatic Cancer, 4) which is lost in 50% of pancreatic cancers [18]. 

A characteristic of pancreatic cancer is the severe desmoplastic reaction in the stroma surrounding the neoplastic cells that is usually responsible for its low neoplastic cellularity [19]. The pancreatic stellate cells (also known as myofibroblasts) have a critical role in the formation and turnover of the stroma. Indeed, these cells secrete collagen and other components of the extracellular matrix upon activation by growth factors and thus contribute to the fibrotic/hypoxic milieu that is characteristic of this tumor [20]. Recent evidence suggests that the stroma might be considered as a dynamic compartment rather than a mechanical barrier, which is involved in the process of tumor formation, progression, invasion, and metastasis [19, 21]. Furthermore, stromal cells express multiple proteins such as SPARC (secreted protein, acidic, cysteine rich) and hedgehog path-way elements that have been associated with poor prognosis and resistance to treatment [22, 23]. 

In addition a group of cancer cells with stem-cell properties has been identified within the tumor [24, 25]. These cells represent a small fraction of the neoplastic cells (1-5%) and are shown to be resistant to chemo and radiation therapy, which, in turn, may explain why these treatments have poor and transient efficacy. 

It is well known that invading and metastasizing cells from epithelial tumours may display characteristics of mesenchymal cells, thus suggesting a transition from an epithelial to a mesenchymal cell phenotype during cancer progression [26]. This epithelial-to-mesenchymal transition (EMT) is associated with morphological as well as molecular changes that enable tumor cells to migrate from the tissue of origin and metastasize to distant sites [26]. Common established markers of EMT in cancer tissues are: loss of E-cadherin and gain of N-cadherin and vimentin expression, as well as an increased expression of the EMT-inducing transcription factors Snail, Slug, Twist, ZEB1, and ZEB2 [27]. However, available data on EMT in pancreatic cancer are not always consistent [28-32]. For instance, Nakajima and colleagues showed that N-cadherin expression in pancreatic cancer tissues correlated with neural invasion, although no inverse correlation between E-cadherin and N-cadherin expression was detected [31]. At variance with this study, Cates *et al.* showed no N-cadherin expression in pancreatic cancer [28]. The observed discrepancy between the results of different studies may be due at least in part to the heterogeneity of patient populations, as well as to the different methodological approaches. Noteworthy, *Rhim et al.* have recently demonstrated in a mouse model of pancreatic cancer that cells from low-grade PanINs are able to breach basement membrane and reach the circulatory system [33]. These cells had the appearance of mesenchymal cells and express markers commonly associated with EMT (such as Snail and ZEB1). The presence of such cells was also independently confirmed in some samples of human PanIN and therefore represents an in vivo example of the “long-sought” EMT.

## MOLECULAR MARKERS FOR DIAGNOSIS

Several markers have been proposed for diagnosis of pancreatic cancer (Table **2**).

The most commonly used marker for pancreatic cancer is CA19-9 or sialylated Lewis (a) blood group antigen. The sensitivity of CA19-9 is ~80% and the specificity is ~90% [34, 35]. However, the use of this marker as diagnostic tool has some limitations [34-37]:

- Elevated CA19-9 levels are often observed in benign obstructive juandice, chronic pancreatitis, liver cirrhosis, and cholangitis.
- Increased levels of CA19-9 are observed in multiple types of adenocarcinomas, especially in advanced gastrointestinal cancers.
- CA19-9 is not expressed in subjects with Lewis a- b- genotype.
- Lack of CA19-9 sensitivity for early or small-diameter pancreatic cancer (< 3 cm).
- Poorly differentiated pancreatic cancers also appear to produce less CA19-9 than either moderately or well-differentiated cancers.

Despite these limitations, CA19-9 is the single most useful blood test in differentiating benign from malignant pancreatic disorders to date. Efforts have been made to either improve the diagnostic ability of CA19-9 or to develop tumor markers that can supplant serum CA19-9 in the diagnosis of asymptomatic pancreatic neoplasms. For istance, Xue and colleagues report of a simplified diagnostic panel of CA19-9, ApoC-I and ApoA-II that improve the diagnostic ability of CA19-9 alone [38]. Furthermore, recent studies showed the potential diagnostic significance of alpha-enolase (ENOA) [39, 40]. Indeed, ENOA is up-regulated in pancreatic cancer and elicits the production of auto-antibodies that were shown to complement the diagnostic performance of serum CA19.9 levels in both advanced and resectable pancreatic cancer [39]. At the same time, global gene expression studies have pointed out several candidate genes, whose secreted products have shown potential as new serum markers for pancreatic cancer. Among others, promising candidates were identified: MIC1 (macrophage inhibitory cytokine 1), osteopontin, tissue inhibitor of matrix metalloproteinase-1, and mesothelin genes [41-46]. None of these have been shown to be superior to CA19-9 and none are widely used. Large-scale proteomic analysis of pancreatic cancer has pointed to a set of candidate biomarkers [47-49]. These studies were mainly focused on the analysis of cancer “secretome”, and all evidenced the need to identify a panel of serum marker rather than a single molecule to achieve the sensitivity and specificity for screening asymptomatic subjects for pancreatic cancer. Among others, perlecan, CD9 and fibronectin receptors were identified as promising circulating tumor marker [49]. Pancreatic juice maybe considered as a valuable source of tumor biomarkers [50, 51]. Mutant *KRAS*, for example, is readily detected in pancreatic juice but can be identified in the circulation only at the stage of non-resectable pancreatic cancers [52]. The development of juice-based markers for the screening in the general population is, however, limited by the invasive procedure necessary to obtain pancreatic juice. On the other hand it is possible to envision the use of juice-based markers in high-risk subjects suspected to harbour early-stage pancreatic cancer. Furthermore, different quantitative assays (such as quantitative methylation-specific polymerase chain reaction or real-time polymerase chain reaction) have been used to explore the pancreatic juice searching for novel pancreatic cancer markers [53, 54]. Despite the promising results of these studies, the real performance of these quantitative techniques needs to be assessed in large-scale studies. 

The diagnostic significance of tissue-based markers has been also investigated in pancreatic cancer. As mentioned above, detection of *KRAS* mutations in different body fluids has been addressed using different techniques. Mutations of *KRAS* gene are present in 75-90% of pancreatic cancers and appear to play a role in the early stages of carcinogenesis [55]. The diagnostic ability of *KRAS* is limited by the lack of specificity and sensitivity since mutations can occur in several pathological conditions other than pancreatic cancer. Mucins are membrane proteins largely investigated as tumor markers, and particularly MUC1 and MUC4 have been implicated in pancreatic carcinogenesis [56]. The potential of mucins to differentiate pancreatic cancer from benign pancreatic tissue was investigated by Wang *et al.* [57] that showed as the measurement of MUC1 and MUC5AC can aid cytology in the diagnosis of pancreatic cancer. MicroRNAs (miRNAs) have also been investigated for their ability to aid diagnosis. MiRNAs are an highly conserved family of small RNA molecules (18-24 nucleotides) that regulate the stability and the translational efficiency of mRNA with complementary sequences [58]. Several studies demonstrated the involvement of miRNAs in the regulation of cellular processes such as proliferation and apoptosis, as well as their altered expression in cancers [59]. A characteristic expression profile for miRNA in pancreatic cancer has been recently identified including the over-expression of miR-21, -155, -221 and -222 [60-62]. The expression of these RNA species may be used to differentiate benign from malignant pancreatic tissues [63, 64]. For instance, Bloomston *et al*. showed that 21 miRNAs with increased expression and 4 with decreased expression correctly differentiate pancreatic cancer from normal pancreatic tissue in 90% of cases [63]. Moreover, 15 overexpressed and 8 underexpressed miRNAs differentiated pancreatic cancer from chronic pancreatitis, with 93% accuracy. Other tissue-based markers potentially relevant for pancreatic cancer include: p21, Bcl-2 and SMAD4 [65, 66]. 

## MOLECULAR MARKER FOR PROGNOSIS

The prognostic value of CA19-9 measurement in newly presenting patients with pancreatic cancer has been extensively investigated. Indeed, patients with elevated level of CA19-9 had a worse prognosis than those with low level [67]. At the same time, also post-operative levels of this marker were associated with patient outcome [68, 69]. In these studies both the levels of postoperative CA19-9 and a decrease in the level of this marker following surgical resection as well as negative lymph nodes and a low tumor stage were good prognostic factors [68, 69]. CA19-9 is also used in clinic for post-operative surveillance [70] and monitoring of therapy in advanced disease [71]. The serial determination of CA19-9 can detect recurrent disease months before its clinical or radiological evidence [36], and has also been proposed as a means to assess response to systemic therapies [71]. Most, but not all, studies found that declining marker levels following chemotherapy are associated with a better outcome [71, 72]. However, the American Society of Clinical Oncology (ASCO) states that evaluation of CA19-9 alone is not sufficient to provide definite evidence of disease recurrence without confirmation by imaging and/or biopsy [73]. Indeed, imaging procedures may fail to assess tumor response to sistemic therapy due to the extensive desmoplasia and the surrounding inflammatory changes, or based on recent evidence that most targeted therapies are cytostatic rather than cytotoxic. 

Different additional tumor markers were investigated for their prognostic relevance including KRAS, *TP53* and microRNAs (Table **3**). The data about the prognostic utility of KRAS and *TP53* mutations are contrasting, therefore, these genes cannot be recommended at present for clinical use to determine prognosis in patients with pancreatic cancer [65]. As well as being potentially useful in diagnosis, miRNAs may also have prognostic relevance (Table **4**). Indeed, Bloomston *et al*. showed that 6 miRNAs were differentially overexpressed in long-term survival patients [63]. Furthermore miR-196a-2 was found to predict poor survival (median, 14.3 months [95% confidence interval, 12.4-16.2] *vs*. 26.5 months [95% confidence interval, 23.4-29.6], *P=*0.009). Furthermore, a very recent study showed that patients with high expression of miR-200c had significantly better survival rates than those displaying low level of expression [74]. A plasma miRNAs profiling has been also proposed as a good biomarker assay for pancreatic cancer since the elevated plasma expression of miR-196a and miR-155 was observed in the parallel progression of disease. This assay showed a sensitivity of 64% and a specificity of 89% [75]. 

Recent data from a genome-wide association study (GWAS) on 650 pancreatic cancer patients showed that a single nucleotide polymorphism rs1233556 in the *SSH *genes was associated with decreased desmoplasia and increased overall survival (McWilliams RR, *et al*. 2010 ASCO Gastrointestinal Cancers Symposium. Abstract126). The results of this study suggest that germline mutation of *SSH* may serve as a good prognostic factor and also enforce the idea of targeting SHH pathway in pancreatic cancer. 

Another potential prognostic marker is Caveolin-1 (Cav-1). This protein belongs to the family of caveolins that are membrane proteins involved in signal transduction and tumorigenesis [76]. Very recent data presented at ASCO Symposium revealed that Cav-1 was up-regulated in pancreatic cancer specimens (Williams TM, *et al*. 2010 ASCO Gastrointestinal Cancers Symposium. Abstract 140). Moreover, poorly differentiated carcinomas showed higher level of Cav-1 compared with well-differentiated ones, and further protein overexpression directly correlated to pre-operative CA19-9 levels. The statistical analysis also showed a trend for Cav-1 expression and shorter time to progression. 

## PREDICTIVE MARKERS

Despite remarkable clinical results obtained with targeted agents, many of them failed to prove effective in pancreatic cancer treatment. At the moment, evidence of efficacy exists only for few chemotherapeutics, and, among them, gemcitabine alone or in combination (with capecitabine or platinum derivatives) represents the treatment of choice for patients with advanced inoperable disease [55, 77]. 

**Fig. (4):**
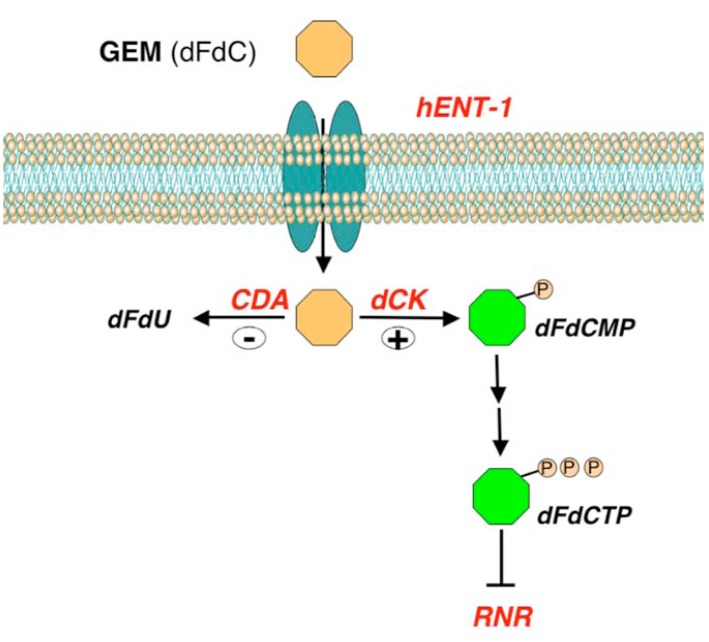
**Gemcitabine metabolism and mechanism.** Simplified representation of the metabolism and mechanism of action of gemcitabine (GEM, dFdC). Gemcitabine enters into the cells mainly through the human equilibrative nucleoside transporter-1 (hENT-1). Inside the cell GEM is activated by the deoxycitidine kinase (dCK) into gemcitabine monophosphate (dFdCMP), then converted in gemcitabine triphosphate (dFdCTP) that, in turns, inhibits the enzyme ribonucleotide reductase (RNR). The inactivation of gemcitabine inside the cells is mainly due to the enzyme cytidine deaminase (CDA) that converts gemcitabine into 2’-deoxy-2’,2’-difluorouridine (dFdU).

Since objective responses to gemcitabine are rare, mechanisms of resistance and markers predictive of response are of particular interest. Gemcitabine is a fluorine-substituted deoxycytidine analog that enters into the cells mainly via the human equilibrative nucleoside transporter-1 (hENT-1) (Fig. **4**). Inside the cell, gemcitabine is activated by the deoxycitidine kinase enzyme (dCK) and inactivated by cytidine deaminase (CDA). The active form of gemcitabine inhibits the enzyme ribonucleotide reductase leading to a decreased level of deoxyribonucleotides essential for DNA synthesis. Ribonucleotide reductase enzyme is composed of three subunits, RRM1, RRM2, and p53R2. Polymorphism of CDA was previoulsy associated with descreased clearance and increased toxicity of the drug [78]. As for other antineoplastic agents, neoplastic cells may develop resistance to gemcitabine mainly through a decreased uptake, an increased inactivation or impairment in the activation of the drug. Preclinical data showed that gemcitabine-resistant cell lines display high level of *hENT-1*, *RRM1*, *RRM2*, and low level of *dCK*. Furthermore, the ratio of *hENT-1 *and *dCK *to *RRM1*, *RRM2 *may predict gemcitabine resistance [79]. Higher RRM1 expression in pancreatic biopsy specimens may predict clinical resistance to gemcitabine [80]. Other studies showed association between high expression of *hENT-1 *with improved clinical outcome in pancreatic cancer patients treated with gemcitabine [81, 82]. The predictive and the prognostic value of hENT-1 and RRM1 still remain to be elucidated. In line with this, a recent study presented at the ASCO Symposium tried to assess the potential prognostic value of hENT-1 and RRM1 in early-resected pancreatic cancer (Schultz NA, *et al.* Abstract N° 169). The analyses were conducted on 84 patients, 18 of which received adjuvant gemcitabine. The results showed that high *hENT-1 *gene levels associated with better overall survival (P= 0.007) and progression free survival (P=0.016). The independent prognostic significance of *hENT-1 *was confirmed in multivariate analysis. No prognostic value was demonstrated for *RRM1, *although, as expected, patients with low RRM1 levels were likely to benefit from adjuvant chemo-therapy. Also microRNAs were investigated as predictive markers of response to gemcitabine and, among these, the most promising candidate is miR-21 (Table **4**) [83]. Indeed, low expression of miR-21 has been associated with benefit from adjuvant treatment in two independent cohorts of pancreatic cancer cases. 

Very recently, *Collison et al.* have re-evaluated the role of EGFR and KRAS in response to gemcitabine and erlotinib, respectively, in light of gene expression profiles [84]. Specifically, starting with data from 66 microdissected pancreatic cancer specimens, they could classify tumors into three groups by a gene signature of 62 differentially regulated genes. The same signature classified 11 cell lines into two groups: “classical” or “quasi-mesenchymal”. Interestingly, classical cell lines were more sensitive to erlotinib while quasi-mesenchymal cells were more sensitive to gemcitabine, suggesting that, despite the almost ubiquitous *KRAS* mutation, EGFR may be more or less influent depending on the tumor molecular subtype and the whole pathway modulation should be considered, especially at its relevant “checkpoints”. This idea was also supported by a previous work by Jimeno *et al.*: by profiling xenografted pancreatic cancers treated with the EGFR inhibitor erlotinib, they proposed a molecular signature for sensitivity to EGFR inhibitor involving core components of the EGFR pathway [85].

## Figures and Tables

**Table 1.** Staging of Pancreatic Cancer

Stage	Tumor†	Nodal Status†	Distant Metastases†	Annotations
IA	T1	N0	M0	Tumor limited to the pancreas, ≤ 2 cm in longest dimension
IB	T2	N0	M0	Tumor limited to the pancreas, ≥ 2 cm in longest dimension
IIA	T3	N0	M0	The tumor extends beyond the pancreas, but the tumor does not involve the major arteries or veins near the pancreas
IIB	T1,T2,T3	N1	M0	The cancer has spread to regional lymph nodes
III	T4	N0 or N1	M0	The tumor extends beyond the pancreas into major arteries or veins near the pancreas. A T4 tumor is unresectable
IV	any T	N0 or N1	M1	There is metastasis to another part of the body, including distant lymph nodes. Distant spread of pancreatic cancer occurs mainly in the liver, peritoneum (lining of the abdominal cavity), and lungs

† T describes the size and location of the primary tumour; N refers to regional lymph nodes; M refers to distant metastases

**Table 2.** Proposed Diagnostic Markers for Pancreatic Cancer

Marker	Reference
MIC1	[42, 44]
OPN	[43]
MSLN	[41]
TIMP-1	[46]
Proteomics	[49]
KRAS (pancreatic juice)	[52]
Mucins (MUC1, MUC5AC)	[52]
MicroRNAs	[63, 64]
p21	[65, 66]
BCL-2	[65, 66]
SMAD4	[65, 66]

*Abbreviations*: MIC1, macrophage inhibitory cytokine 1; OPN, osteopontin; MSLN, mesothelin; TIMP-1, tissue inhibitor of matrix metalloproteinase-1.

**Table 3.** Potential Prognostic Markers for Pancreatic Cancer

Marker	Reference
CA19-9	[67-73]
KRAS	[65]
*TP53*	[65]
miR-196a-2	[63]
miR-200c	[74]
miR-196a, miR-155	[75]
Caveolin-1 (Cav-1)	[76]

**Table 4.** MicroRNAs as Prognostic and Predictive Markers for Pancreatic Cancer

MicroRNA	Expression	Characteristics
miR-452, 105,127, 518a-2, 187, 30°-3p [63]	Up-regulation	Distinguish long-term survivors within patients with node positive disease
miR-196a-2 [63]	Up-regulation	Predicts poor survival
miR-200c [74]	Up-regulation	Better survival rates
miR-196a, 155 (plasma) [75]	Up-regulation	Elevated level associate with disease progression
miR-21 [83]	Down-regulation	Benefits from adjuvant treatment

## FUTURE DIRECTIONS

Future research in pancreatic cancer should focus on the discovery of biomarkers for early diagnosis as well as of markers that can help to select patients likely to respond to specific therapies. Recent advances in the understanding of the biology of this cancer and the availability of preclinical models that recapitulate the complexity of this disease pave the way to new perspectives in the identification of novel markers and targets. For instance, the recognition that pancreatic cancer is a complex entity that include also stromal cells and cancer stem cells led to the development of novel agents with promising anti-cancer activity [23, 24, 85].

Furthermore, a very recent study based on the use of new sequencing technologies has represented a Pandora’s vase for what concerns the abnormalities of pancreatic cancer and hence can be exploited as a launching pad for novel discoveries [86-88]. The results of this study highlighted that each tumor harbours an average of 63 genetic alterations. These alterations involved members of 12 different pathways and processes. In particular, 6 different pathways resulted to be altered in 100% of pancreatic cancers due to mutations affecting at least one of their different components. These pathways are: KRAS-MAPK, Apoptosis, G1-S transition, Hedgehog, TGF and Wnt/Notch. Interestingly, they also identified over 500 genes that were overexpressed in the vast majority of cancers. Among them, more than a half encoded for membrane or secreted proteins, thus providing a source for the identification of novel markers/targets for pancreatic cancer. Beyond this seminal work, other recent studies performed massive-scale sequencing analysis to uncover pancreatic cancer heterogeneity and address the clonal relationship among primaries and metastasis [87, 88]. The approach they used, referred as comparative lesion sequencing, clearly indicated a high degree of genetic complexity and heterogeneity among metastatic deposits that substantially reflect that of the primaries from which they derived. At the same time, intriguing insights into temporal patter of tumor clonal evolution have indicated a huge time-window to diagnose pancreatic cancer before metastatic spread occurs. Despite far from identifying all the pre-requisite events that trigger metastatic dissemination, findings from these studies have important clinical implication both from a diagnostic and therapeutic point of view as metastasis represents the most deadly feature of pancreatic cancer. Altogether, comprehensive genome-wide studies revealed that beyond known and well characterized alterations, a plethora of different molecular features defined a very complex and heterogeneous disease. When ignored, molecular heterogeneity can lead to failures in therapeutic treatments, as drugs that may have efficacy in subgroups of patients with specific molecular phenotypes may show marginal response when tested in a large group of unselected patients. Although far from being introduced into a routine clinical setting, findings from these studies indicate a new perspective through which we look at the clinical management of pancreatic cancer towards a personalized cancer medicine. In line with this, efforts in this direction have been done recently by using somatic alterations annotated by massively sequencing of cancer primary samples to monitor residual and recurrent disease in blood sample from the same individuals [89]. This approach, termed PARE (Personalized Analysis of Rearranged Ends), was shown to be a highly sensitive clinical tool for colorectal and breast cancer, but it is obviously applicable in other tumor types including pancreatic cancer.

## SUPPORTS

This work is based on studies supported by Associazione Italiana Ricerca Cancro (AIRC), Milan, Italy; Fondazione CariPaRo, Padova, Italy; Fondazione Cariverona, Verona, Italy; Ministero della Salute, Rome, Italy.
